# Self-Compassion and Cultural Values: A Cross-Cultural Study of Self-Compassion Using a Multitrait-Multimethod (MTMM) Analytical Procedure

**DOI:** 10.3389/fpsyg.2018.02638

**Published:** 2018-12-21

**Authors:** Jesus Montero-Marin, Willem Kuyken, Catherine Crane, Jenny Gu, Ruth Baer, Aida A. Al-Awamleh, Satoshi Akutsu, Claudio Araya-Véliz, Nima Ghorbani, Zhuo Job Chen, Min-Sun Kim, Michail Mantzios, Danilo N. Rolim dos Santos, Luiz C. Serramo López, Ahmed A. Teleb, P. J. Watson, Ayano Yamaguchi, Eunjoo Yang, Javier García-Campayo

**Affiliations:** ^1^Primary Care Prevention and Health Promotion Research Network, RedIAPP, Zaragoza, Spain; ^2^Department of Psychiatry, University of Oxford, Oxford, United Kingdom; ^3^School of Psychology, University of Sussex, Falmer, United Kingdom; ^4^Department of Psychology, University of Kentucky, Lexington, KY, United States; ^5^School of Physical Education, University of Jordan, Amman, Jordan; ^6^Graduate School of International Corporate Strategy, Hitotsubashi University Business School, Hitotsubashi University, Hitotsubashi, Japan; ^7^School of Psychology, Universidad Adolfo Ibáñez, Santiago, Chile; ^8^Department of Psychology, University of Tehran, Tehran, Iran; ^9^Department of Psychology, Clemson University, Clemson, SC, United States; ^10^Department of Communicology, University of Hawai‘i at Mānoa, Honolulu, HI, United States; ^11^Department of Psychology, Birmingham City University, Birmingham, United Kingdom; ^12^Laboratório de Ecologia Comportamental e Psicobiologia (DSE/CCEN), Universidade Federal da Paraiba, João Pessoa, Brazil; ^13^Special Education Department, Faculty of Education, King Khalid University, Asir, Saudi Arabia; ^14^Department of Psychology, The New Valley Faculty of Education, Assiut University, Assiut, Egypt; ^15^Department of Psychology, University of Tennessee at Chattanooga, Chattanooga, TN, United States; ^16^College of Community and Human Services, Rikkyo University, Saitama, Japan; ^17^Department of Psychology, Korea University, Seoul, South Korea; ^18^Instituto de Investigación Sanitaria Aragón, Hospital Universitario Miguel Servet, Zaragoza, Spain

**Keywords:** self-compassion, SCS, cross-cultural, multitrait-multimethod, MTMM, CFA

## Abstract

Self-compassion is natural, trainable and multi-faceted human capacity. To date there has been little research into the role of culture in influencing the conceptual structure of the underlying construct, the relative importance of different facets of self-compassion, nor its relationships to cultural values. This study employed a cross-cultural design, with 4,124 participants from 11 purposively sampled datasets drawn from different countries. We aimed to assess the relevance of positive and negative items when building the self-compassion construct, the convergence among the self-compassion components, and the possible influence of cultural values. Each dataset comprised undergraduate students who completed the “Self-Compassion Scale” (SCS). We used a confirmatory factor analysis (CFA) approach to the multitrait-multimethod (MTMM) model, separating the variability into self-compassion components (self-kindness, common humanity, mindfulness), method (positive and negative valence), and error (uniqueness). The normative scores of the Values Survey Module (VSM) in each country, according to the cultural dimensions of individualism, masculinity, power distance, long-term orientation, uncertainty avoidance, and indulgence, were considered. We used Spearman coefficients (*r*_s_) to assess the degree of association between the cultural values and the variance coming from the positive and negative items to explain self-compassion traits, as well as the variance shared among the self-compassion traits, after removing the method effects produced by the item valence. The CFA applied to the MTMM model provided acceptable fit in all the samples. Positive items made a greater contribution to capturing the traits comprising self-compassion when the long-term orientation cultural value was higher (*r*_s_ = 0.62; *p* = 0.042). Negative items did not make significant contributions to building the construct when the individualism cultural value was higher, but moderate effects were found (*r*_s_ = 0.40; *p* = 0.228). The level of common variance among the self-compassion trait factors was inversely related to the indulgence cultural value (*r*_s_ = -0.65; *p* = 0.030). The extent to which the positive and negative items contribute to explain self-compassion, and that different self-compassion facets might be regarded as reflecting a broader construct, might differ across cultural backgrounds.

## Introduction

Compassion has been described as an orientation of mind that recognizes pain, the universality of pain and the capacity to meet pain with empathy and kindness (Feldman and Kuyken, 2011). Compassion for the self (or self-compassion) is this attitude focused on the self. It has been defined as “being touched by and open to one’s own suffering, not avoiding or disconnecting from it, generating the desire to alleviate one’s suffering and to heal oneself with kindness,” and involves “offering non-judgmental understanding to one’s pain, inadequacies and failures, so that one’s experience is seen as part of the larger human experience” (Neff, 2003a,b). Psychological understanding of self-compassion regards all its features as co-occurring to form a particular orientation of mind, that is framed in a motivational system focused on an attentional sensitivity to suffering and a commitment to relieve it by the recognition of the universality of pain in human experience, and also the capacity to meet that pain with equanimity (Feldman and Kuyken, 2011; MacBeth and Gumley, 2012).

The issue of whether the different elements of self-compassion hang together to form an overarching construct is important, both theoretically and practically. This is because we need to understand the construct and, based on that understanding, how best to train and cultivate it. However, it is unclear to what extent the different dimensions of self-compassion co-occur in the general population and indeed across different cultural contexts. In this paper, we will explore this issue with particular reference to self-compassion as assessed by the “Self-Compassion Scale” (SCS) (Neff, 2003a). This measure is the most used approach to assess self-compassion up to now, and operationalizes it as comprised of three inter-related general traits: “self-kindness” or being kind rather than judgmental toward the self; “common humanity” which describes seeing one’s suffering as part of the human condition, rather than as isolating; and “mindfulness” which consists of the capacity to hold painful feelings mindfully, rather than being over-identified with them (Neff, 2003b).

Many studies have examined the factor structure of the SCS. Originally, a three factor correlated structure, incorporating the dimensions of self-kindness, common humanity and mindfulness, was proposed theoretically (Neff, 2003b). However, evidence for this structure is marginal, and research has more frequently identified a six-factor correlated model in which the positively and the negatively valenced items on each of the theoretical dimensions form distinct factors (self-kindness vs. self-judgement; common humanity vs. isolation; mindfulness vs. over-identification) (Neff et al., 2008; Lee and Lee, 2010; Hupfeld and Ruffieux, 2011; Azizi et al., 2013; Arimitsu, 2014; Garcia-Campayo et al., 2014; Petrocchi et al., 2014; Williams et al., 2014; Mantzios et al., 2015; Bento et al., 2016; de Souza and Hutz, 2016). A single second-order factor of “self-compassion,” in addition to the referred six first order-factors, has also been proposed (Neff, 2003b), and this structure has been obtained in a number of studies (e.g., Chen et al., 2011; Castilho et al., 2015; Benda and Reichová, 2016; Dundas et al., 2016). It has been argued that whilst a six-factor correlated model provides the best fit across a range of samples, a bi-factorial model, in which a general self-compassion factor is derived alongside six separate group factors, provides a reasonable fit in non-clinical samples (e.g., Kotsou and Leys, 2016; Tóth-Király et al., 2017; Veneziani et al., 2017; Cleare et al., 2018). The six-correlated factors model, single bi-factor model and also a two-correlated bi-factor model have recently been observed, but using six subscale scores—representing the extreme but independent poles of the original dimensions—or a total overall score—as a single dimension that summarize the total construct—has been recommended (Neff et al., 2018).

A problem emerging from research demonstrating conflicting factor structures of the SCS relates to the relative contribution of positively and negatively valenced items to the theoretical components of self-compassion. The issue of whether the negatively valenced items of the SCS genuinely reflect the absence of self-compassion, has been raised by several studies that have identified factor solutions in which the negative items load together (e.g., López et al., 2015; Costa et al., 2016; Pfattheicher et al., 2017). Other researchers have recommended the use of two independent subscale scores to capture the positive and negative group factors, advising against of the estimation of a single total score (Brenner et al., 2017; Coroiu et al., 2018; Halamová et al., 2018). A study by Zeng et al. (2016) even identified the three positive facets (self-compassion, mindfulness, common humanity) alongside a general factor representing all negative aspects grouped. These studies and others have led to the proposal that in some contexts, treating the negatively valenced items on the SCS as a single higher order “self-criticism” or “self-coldness” factor—which correlates highly with distress and psychopathology—would be justified (Mills et al., 2007; Montero-Marin et al., 2016a; Muris and Petrocchi, 2017; Brenner et al., 2018; Muris et al., 2018). Whether this factor could be meaningfully regarded as measuring variance related to the construct of self-compassion rather than some broader negative trait or response tendency or bias (Chen, 2008), is unclear. Thus, research to date is equivocal regarding whether it is meaningful to think of self-compassion as a single trait when using the SCS. One possible explanation for the conflicting factor structures of the SCS identified in the literature is that the extent to which the various theoretical components of self-compassion (self-kindness–self-judgement, common humanity–isolation and mindfulness–over-identification) converge, may differ between individuals and across contexts (e.g., cultural, organizational), with components aggregating or disaggregating as a function of differences in contextual cultural values, learning histories, the extent of the deliberate cultivation of related skills, or divergence in conceptualizations of item meaning.

It has previously been proposed that overall levels of self-compassion may be at least partially culturally determined (e.g., Neff et al., 2008), and that self-compassion might be a context-dependent characteristic influenced by group norms, values and practices (Gilbert et al., 2011). It would also be possible that cultural values and practices affect the underlying structure of the self-compassion construct depending on whether a culture is prone to see self-compassion through the lens of positively and/or negatively valenced facets. For instance, it has been suggested that some cultural frameworks, such as those which emphasize self-improvement through a self-critical mind-set and the practice of shaming in response to failure or transgression, may be associated with high levels of negative self-referent emotions, and a relative absence of self-compassion (Neff et al., 2008). The mean level of a component—e.g., self-criticism—observed within a group, is not the same as the centrality of this component in explaining a broad construct—e.g., self-compassion. However, one possibility might be that the relative contribution of the positively and negatively valenced items to the self-compassion construct may differ cross-culturally at least partly as a function of the extent to which—among others—negative feelings toward the self, such as shame and guilt, are used to control or regulate behaviors (Watson et al., 2010; Gilbert et al., 2011). In other words, self-compassion might be best captured by items that tap into positive or negative facets of the construct, depending on the extent to which contextual influences emphasize the positive or negative aspects of self-compassion. Another possibility is that in cultures which emphasize self-compassionate action as a feature of spiritual practice, the different aspects of the self-compassion construct may be more closely associated with one another than in those cultures that do not emphasize these practices. That is, one would expect a high convergence among the self-compassion facets when a cultural context is consistently influencing and promoting the expression of all related aspects of self-compassion in the same direction.

A comprehensive and largely studied potential frame for conceptualizing relevant cultural differences that might shape self-compassion is the Cultural Dimensions Theory of Hofstede (2001), Hofstede et al. (2010). According to Hofstede, culture can be understood as the collective mental programming of the human mind that distinguishes the members of one group, shapes the values of group members, and through these values influences behavior. Six cultural dimensions are proposed by this theory: individualism-collectivism, masculinity-femininity (i.e., task- vs. person-focussed orientation), power distance, long-term orientation, uncertainty avoidance, and indulgence-restraint. Individualism expresses the preference for a social framework in which individuals do not expect their relatives to look after them, and individual choices are expected. Masculinity represents a preference for achievement, material rewards and success in a competitive way. Power distance refers to the degree to which the less powerful members of a society accept that power is distributed unequally. Long-term orientation is a pragmatic point of view that encourages planning and education as a way to prepare for the future. Uncertainty avoidance expresses the levels of discomfort the members of a society feel when coping with ambiguity. Indulgence corresponds to societies that allow gratification of basic human drives related to enjoying life, having fun, and acting on impulses.

It is possible to suppose distinct potential associations between these cultural values and self-compassion—e.g., individualism could be associated with more prominent negative, competitive and isolating forms of interpersonal relating (Gilbert, 2014), giving more prominence to the negatively worded self-compassion items. Likewise indulgence may drive to open and disaggregate the interpretation of self-compassion to the point of not having all the facets aligned to self-care elements (Mantzios and Egan, 2017), and thus lowering their factor convergence. Despite these speculations we argue it is premature to articulate directional hypotheses. So far, it is difficult to make further strong claims for none of the cultural values in the way through which they could specifically be determining the structure of the construct under study, so we propose an exploratory and hypothesis generating approach.

To date most studies of self-compassion have evaluated different SCS structures by using distinct analytical models searching for the best fit to the data. However, they have not considered the extent to which differences in factor structures might be attributed to distinct response tendencies across samples, either in the relative contribution of positively and negatively valenced items to each component or the degree of inter-relationship between the different components of self-compassion, after controlling for possible method effects. In view of this, the aim of this study was not to test the SCS factorial structure—something that has been repeatedly done—but to separate the variance coming from the general self-compassion components of self-kindness, common humanity and mindfulness, and the variance coming from the corresponding positively and negatively valenced items, as well as to assess the degree of association between the referred self-compassion components, for the purpose of evaluating the possible influence of cultural values. We studied a number of samples, keeping some population characteristics similar (e.g., approximate age, and educational level) but varying cultural backgrounds. In order to evaluate the possible influence of cultural values on the self-compassion construct, we drew on the dimensions described by Hofstede et al. (2010) cultural values model. The analysis was exploratory, with the intention of building theory that can be developed in future studies, but it was driven by the question of whether/to what extent some cultural distinctions may be related to differences in the self-compassion construct structure. The heuristic hypothesis was that samples drawn from different cultural contexts and values might differ in the relative contribution of positive and negative items and the degree of common variance among the general self-compassion components, and that these differences might relate in meaningful ways to the dominant cultural values of each context.

## Materials and Methods

A cross-sectional and cross-cultural design was adopted, using self-report data on the SCS and the norm scores on cultural values of the countries included in the study, referred to below.

### Participants

In total data from 4,124 participants, coming from 11 independent samples and 5 pairs of distinct geographical areas, were included. To create a comparable and relatively homogeneous overall sample, all participant samples were undergraduate university students, drawn purposively from different parts and cultural areas of the world. We tried to identify samples of sufficient size to allow for the recommended 10:1 ratio for the number of participants to the number of test items included in the multitrait-multimethod (MTMM) structural equation model (SEM), providing psychometric adequacy to the analysis (Kline, 1998). Sample sizes ranged from *n* = 238 (Iran) to *n* = 570 (Spain). The overall mean age of participants was 20.98 years (SD = 2.58), and there were 2,726 (66.1%) females and 1,398 (33.9%) males. The general characteristics of each particular sample included in the present study can be seen in Table 1.

**Table 1:** Characteristics of the study samples.

Country	Language	Cultural area	*n*	Studies	Females^†^	Age^‡^
Brazil	Portuguese	South America	456	Various disciplines	282 (61.8%)	23.43 (5.61)
Chile	Spanish	South America	274	Psychology	187 (69.7%)	20.53 (3.50)
Greece	Greek	Mediterranean	359	Various disciplines	331 (92.2%)	20.42 (2.23)
Spain	Spanish	Mediterranean	570	Health careers	354 (62.1%)	21.87 (3.83)
United Kingdom	English	Anglospere	362	Various disciplines	340 (93.9%)	19.98 (2.04)
United States	English	Anglospere	356	Psychology	244 (68.5%)	18.65 (2.78)
Iran	Farsi	Islamics	238	Various disciplines	113 (47.5%)	21.55 (2.35)
Saudi Arabia	Arabic	Islamics	373	Various disciplines	180 (48.3%)	19.96 (0.70)
Egypt	Arabic	Islamics	272	Education	144 (52.9%)	19.79 (0.73)
Korea	Korean	Far East	313	Various disciplines	159 (50.8%)	24.62 (1.95)
Japan	Japanese	Far East	551	Communication	392 (71.2%)	19.92 (2.65)

*^†^Frequencies (percentages). ^‡^Means (SDs).*

### Procedure

Considering that a country does not necessarily equate to a particular culture or cultural value *per se*, and to gather a sufficient range of cultural diversity and geographical dispersal to be able to establish meaningful comparisons, we contacted researchers from 11 different countries coming from 5 great geographical regions around the world, with distinct languages and historical backgrounds. They included South America (Chile: Spanish; Brazil: Portuguese), the Anglosphere (United Kingdom: English; US: English), Mediterranean area (Greece: Greek; Spain: Spanish), Islamic countries (Iran: Farsi; Saudi Arabia: Arabic; Egypt: Arabic), and the Far East (Japan: Japanese; Korea: Korean). The Brazilian, Greek, United Kingdom, and Spanish samples represent previously unpublished data. The other samples were drawn in part, as secondary data, from the following previously published works: Chile (Araya et al., 2017), Iran (Ghorbani et al., 2012), Japan (Yamaguchi et al., 2014), Korea (Hwang et al., 2016), Egypt and Saudi Arabia (Teleb and Al-Awamleh, 2013), and United States (Watson et al., 2010).

### Measurements

#### Self-Compassion

To measure “self-compassion,” we used the SCS in its long form (Neff, 2003a). The SCS long form is a 26-item questionnaire designed to assess self-compassion across the facets of self-kindness and its opposite self-judgment (e.g., “I try to be loving toward myself when I’m feeling emotional pain,” and “I’m disapproving and judgmental of my flaws and inadequacies”); common humanity and its opposite isolation (e.g., “I try to see my failures as part of the human condition,” and “When I’m feeling down, I tend to feel like most other people are happier than I am”); and mindfulness and its opposite over-identification (e.g., “When something upsets me, I try to keep my emotions in balance,” and “When I’m feeling down, I tend to obsess and fixate on everything that is going wrong”). The items assess how respondents perceive their actions toward themselves in difficult times, using a Likert-type scale from 1 (“almost-never”) to 5 (“almost-always”). Data from all samples were based on the following eight validated versions of the 26-item SCS that used translation/back-translation procedures and demonstrated appropriate psychometric properties: the Arabic (Teleb and Al-Awamleh, 2013), Brazilian (de Souza and Hutz, 2016), English (Neff, 2003a), Greek (Mantzios et al., 2015), Japanese (Yamaguchi et al., 2014), Korean (Kim et al., 2008), Farsi (Ghorbani et al., 2013), and Spanish (Garcia-Campayo et al., 2014; Araya et al., 2017) versions.

#### Cultural Values

We used the norm scores of the “Values Survey Module” (VSM)—which has accumulated a large amount of cross-cultural data on cultural values all around the world—to position each country included in the present study according to the dimensions of cultural values of “indulgence,” “individualism,” “masculinity,” “power distance,” “long-term orientation,”, and “uncertainty avoidance” (Hofstede et al., 2010). Each scale ranges from 0 to 100 points, with 50 points as a mid-level. Scales are interpreted so that the higher the scores, the greater the presence of each cultural trait. The VSM is being widely used to compare culturally relevant values between matched respondent samples from different societies. It takes a country-level perspective, and presents adequate internal consistency scores in each dimension (α > 0.70) when comparisons include at least 10 countries (Hofstede et al., 2010).

### Data Analysis

We described the socio-demographic variables in each sample using means (SDs) and frequencies (percentages), depending of the nature of each variable.

**FIGURE 1:**
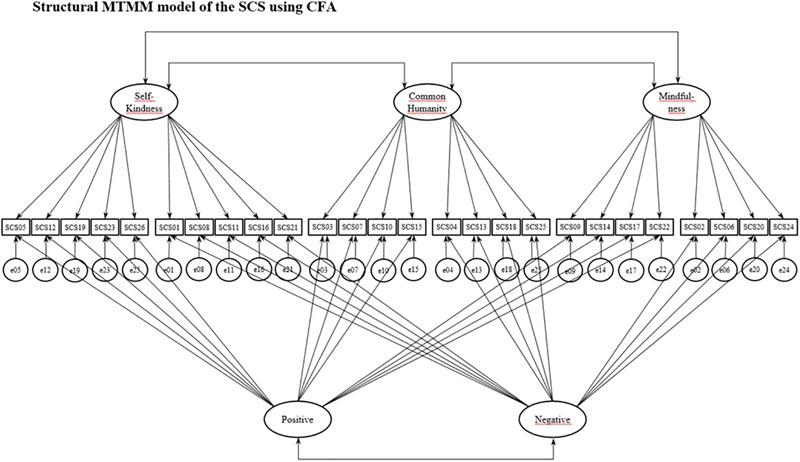
Structural MTMM model of the SCS using CFA. The circles represent latent components, and the rectangles are observable variables (SCS items). One-way arrows represent factor loadings, and two-way arrows are covariances.

In order to evaluate the potential sources of variability of the SCS components across samples, we used a SEM approach to confirmatory factor analysis (CFA), applied to the MTMM complete model (Marsh and Hocevar, 1983; Kenny and Kashy, 1992). The reason we used the MTMM model by means of standard CFA estimation was because it permits the orthogonal decomposition of the overall variance into trait (T), method (M) and uniqueness (U), allowing estimation of the relationships among trait factors and also between method factors. In the CFA specification of the MTMM complete model, each measured variable is considered to be a function of trait, method, and unique factors; that is, each item serves as an indicator on both a single trait factor and a single method factor plus the unique term. The method factor was conceived as the positive and negative valence of the items. Thus, the proposed model includes three general correlated self-compassion trait factors (self-kindness, common humanity and mindfulness), and two correlated method factors (positive and negative valences) (Figure 1).

The original theoretical SCS background assumes the positive and negative facets constitute opposite poles operating within a system of relationships (Neff, 2003b). Thus, we assumed that the method factors were not independent, and therefore we did not use the correlated uniqueness approach because of the potential problem of inflation, which overestimates convergence and worsens discriminant validity (Marsh, 1989; Kenny and Kashy, 1992). Neither did we use the fixed method model (Kenny and Kashy, 1992) because both method factors were of central interest, and in absence of other self-compassion measures reducing the number of method factors was not feasible. It was also not possible to make the assumption that the method effects sum to 0, because bias due to negative valence of items might not be exactly offset by bias due to the corresponding positive valence. That is, the fit and relations of positive and negative halves might not be the same (Montero-Marin et al., 2016a,b; Muris and Petrocchi, 2017; Muris et al., 2018).

In this context of strong theoretical and methodological restrictions, and in order to facilitate achievement of convergence by defined matrix solutions, we: (a) kept all sample sizes as high as possible, imputing possible missing data by means of linear interpolation in each sample separately (Marsh, 1989); (b) used each SCS item as an indicator of each latent dimension in accordance with the original SCS background (Neff, 2003a) and the MTMM model, in order to maintain a high indicator/factor ratio and thus minimizing the potential problem of equal factor loadings (Kenny and Kashy, 1992)—which can drive to models that cannot be uniquely estimated; (c) we simplified the complete MTMM model including only the trait-trait and method-method inter-factor correlations object of interest, discarding trait-method correlations to facilitate more realistic estimations (Kenny and Kashy, 1992); (d) fixed the factor variance of each latent factor to be 1.00 so that the factor variance/covariance input matrix was a correlation matrix, from which factor loadings were estimated (Marsh, 1989).

Mardia’s multivariate kurtosis statistics were estimated to evaluate items’ distribution, and polychoric correlation matrices, developed for the analysis of relationships between polytomous categorical variables, were calculated to evaluate the relationships among the SCS items. We ensured the adequacy of the matrices by assessing: (a) the corresponding determinants in order to discard possible problems of multi-collinearity; (b) the Kayser-Meyer-Olkin (KMO) index as a measure of sampling adequacy; and (c) Barlett’s test for sphericity to check if there was enough redundancy between the items to be summarized with a smaller number of factors. In view of its robustness, the un-weighted least squares (ULS) was the method used for developing covariance structures and factor extraction. The ULS method does not provide inferential estimations for assessing model-data fit based on the χ^2^ distribution, and therefore, does not provide significance *p*-values for the parameters obtained and does not permit invariance measurement approaches. However, it has the following important advantages: (a) it does not require any previous distributional assumption of data; (b) it is quite robust, and usually converges because of its high efficiency in terms of computation; (c) it tends to supply less biased estimates of the true parameter values than classical methods or than far more complex procedures; (d) it is an appropriate choice for the case of not excessively large samples; (e) it shows good performance when working with polychoric matrices in case of absence of multi-normality; and (f) it tends to provide more accurate estimates even with large models (Briggs and MacCallum, 2003; Lee et al., 2012). Therefore, since our objective at this level was to obtain model parameters that were as robust as possible, regardless of contrast tests of significance, we opted to use the referred ULS method.

We assessed the raw internal consistency of factors by calculating McDonald’s omega (ω) composite reliability values—under the ULS method and using polychoric matrices—which can be interpreted as the square of the correlation between the scale score and the latent variable common to all the indicators in the infinite universe of indicators of which the scale indicators are a subset (McDonald, 1999). This index assumes a congeneric model of reliability, which means that factor loadings are allowed to vary, taking into account the item-specific measurement error and providing a more realistic estimate of true reliability.

From a general perspective, in order to evaluate the MTMM model fit to the data—in addition to the chi-square statistic comparing the tested model and the independence model to the saturated model (CMIN), and also taking into account the number of parameters in the model (NPAR)—we examined the fit indices that the ULS method reports, such as the goodness-of-fit index (GFI), the adjusted goodness-of-fit index (AGFI), the normed-fit index (NFI), Bollen’s relative fit index (RFI), and the standardized root mean square residual (SRMR). GFI and AGFI refer to the explained variance of the proposed model, and although GFI is sensitive to sample size, AGFI corrects this limitation according to the degrees of freedom and the number of variables included in the model. Both indices are considered acceptable when >0.90 (Byrne, 2016). The NFI measures the proportional reduction in the adjustment function when going from the null to the proposed model and is considered acceptable when >0.90 (Levy et al., 2006). RFI takes into account the discrepancy for the model evaluated and for the baseline model, it is very good close to 1, and in general it is considered that the higher the values the better (Bollen, 1986). SRMR is the standardized difference between the observed and the predicted covariance, indicating an appropriate fit for values when <0.08 (Hu and Bentler, 1999). All of these indices are valid for the ULS procedure. Taken together, they provide a reliable evaluation of the solution and additional information regarding absolute and incremental model-data fit assessment.

From an analytical point of view, to separate the true variance on the trait factors of self-kindness (SK), common humanity (CH), and mindfulness (MI), from the variance resulting from the measurement method (positive and negative), we calculated the percentage of explained variance in each of the SCS items. It was estimated as the square of the standardized factor loadings resulting from the underlying trait and the reporting method, so that the unexplained variance was termed uniqueness. To facilitate comparability among samples, we averaged the variance components (e.g., T, M, U) grouping them into the categories of SK+, SK-, CH+, CH-, MI+ and MI- according to the item valence. The variance components of the positive and negative items were also summarized by averaging them separately. The percentage of common variance among factors was calculated by means of the implied determination coefficients (*R*^2^) from the standardized correlations between latent components. The logic is that the more shared variance between the positive and negative method factors, the more dependence exists between the SCS method halves, while the more shared variance among the trait factors, the greater the degree to which they converge on the same construct.

Finally, we estimated the degree of associations between the trait parameters of the MTMM model, free of method effects (e.g., the averaged percentage of explained variance of the positive items and the averaged percentage of explained variance of the negative items, regarding the self-compassion trait factors, as well as the convergence among the self-compassion trait factors by averaging their common variance by means of determination coefficients), and the norm scores of each country in the cultural values of individualism, masculinity, power distance, long-term orientation, uncertainty avoidance, and indulgence. For that, we calculated Spearman’s rho correlation coefficients (*r*_s_) between the referred trait parameters and the cultural values of the countries included in the study. Because the small sample size at the level of countries (*n* = 11), we gave relevance to the strength of relationships by considering effects sizes, with the following intervals for Spearman’s rho coefficients: from 0.10 to 0.30, small effects; from 0.31 to 0.50, intermediate effects; and 0.51 and higher, strong effects. We calculated *p*-values with an alpha level of 0.05, and due to the theory-building exploratory nature of the study, no corrections for multiple measurements were applied. Analyses were performed using STATA-12, SPSS-19 and Amos-7 statistical packages.

## Results

### Matrices, Composite Reliability and MTMM Model Fit

The percentage of imputed missing values was very low, ranging from 0.0% (Brazil, Egypt, Spain, Japan, Korea and Saudi Arabia) to 1.2% (Iran). Mardia’s statistic were moderate and ranged from 3.91 (Egypt) to 74.68 (Japan), although they were significant in all cases, and therefore, the estimation of polychoric correlation matrices was advisable. The raw composite reliability values for the trait and method factors were appropriate, although the Islamic and Far East samples showed rather fair values in some of the trait factors (Table 2). Polychoric matrices showed good KMO indices (except in the case of Egypt, that was a bit fair), and also determinant values (≤0.001). Bartlett’s statistics (*p* < 0.001) revealed adequate properties with which to perform subsequent factorial analyses (see Table 3). The CFA applied to the MTMM model with the three correlated trait factors and the two correlated method factors presented adequate fit in all the samples used (although the Egyptian sample showed rather scarce values). In general terms, the Islamic samples showed the worst CFA fit values, while the United Kingdom sample showed the best fit to the data (Table 3). All of the samples reached well defined solutions in the specific conditions described above, which means that the interpretation of the parameters of the models and their use for the next analyses was legitimate.

**Table 2:** Composite reliability values of factors sorted by country.

Country	SK	CH	MI	Positive	Negative
Brazil	0.81	0.72	0.79	0.85	0.90
Chile	0.88	0.73	0.80	0.87	0.88
Greece	0.91	0.82	0.84	0.92	0.93
Spain	0.85	0.70	0.78	0.86	0.89
United Kingdom	0.94	0.89	0.88	0.94	0.94
USA	0.90	0.77	0.79	0.85	0.89
Iran	0.72	0.63	0.67	0.79	0.82
Saudi Arabia	0.59	0.58	0.60	0.75	0.77
Egypt	0.54	0.50	0.58	0.71	0.75
Korea	0.81	0.65	0.64	0.94	0.94
Japan	0.66	0.72	0.67	0.87	0.86

*Values are composite reliability coefficients (McDonalds’s omega). SK: self-kindness, integrating the positive and negative items. CH: common humanity, integrating the positive and negative items. MI: mindfulness: mindfulness, integrating the positive and negative items. Positive integrates the positive items together. Negative integrates the negative items together.*

**Table 3:** Matrix characteristics and fit of the MTMM model for the SCS.

Country	Det	KMO	Ba	CMIN	NPAR	GFI	AGFI	NFI	RFI	SRMR
Brazil	<0.001	0.91	<0.001	482.55	82	0.985	0.980	0.976	0.971	0.041
Chile	<0.001	0.89	<0.001	515.09	82	0.978	0.971	0.968	0.961	0.052
Greece	<0.001	0.94	<0.001	355.31	82	0.989	0.986	0.987	0.984	0.043
Spain	<0.001	0.91	<0.001	539.19	82	0.985	0.980	0.977	0.972	0.041
United Kingdom	<0.001	0.95	<0.001	292.18	82	0.993	0.991	0.992	0.990	0.037
United States	<0.001	0.90	<0.001	81.70	82	0.983	0.978	0.974	0969	0.046
Iran	0.001	0.77	<0.001	704.10	82	0.944	0.927	0.882	0.857	0.062
Saudi Arabia	0.001	0.72	<0.001	946.99	82	0.940	0.922	0.859	0.830	0.063
Egypt	<0.001	0.59	<0.001	1841.27	82	0.882	0.854	0.742	0.708	0.084
Korea	<0.001	0.92	<0.001	269.44	82	0.990	0.987	0.987	0.985	0.039
Japan	<0.001	0.86	<0.001	2469.68	82	0.970	0.961	0.948	0.937	0.052

*Det, determinant of the matrix; KMO, Kayser-Meyer-Olkin index for sampling adequacy; Ba, *p*-value for the Bartlett’s test for sphericity; CMIN, Chi-square statistic comparing the tested model and the independence model to the saturated model; NPAR, number of parameters; GFI, goodness-of-fit index; AGFI, adjusted goodness-of-fit index; NFI, normed-fit index; RFI, Bollen’s relative fit index; SRMR, standardized root mean square residual.*

### Trait, Method and Uniqueness Variance Components

All the variance components for the SCS items in the MTMM model referred in Figure 1 using CFA can be seen in Additional File S1. To facilitate interpretability we show in Table 4 the item variance components, grouping them according to their superordinate trait (self-kindness, common humanity and mindfulness) and valence (positive and negative). After averaging the variance components of the isolated positive and negative items separately, we observed that positive items showed a higher percentage of explained trait variance (T) than method variance (M)—i.e., the trait effect was stronger than the method effect—in Korea (T = 0.43; M = 0.13; U = 0.44), Spain (T = 0.35; M = 0.10; U = 0.55), United Kingdom (T = 0.35; M = 0.26; U = 0.40), Japan (T = 0.26; M = 0.11; U = 0.63), Saudi Arabia (T = 0.21; M = 0.04; U = 0.75), and Egypt (T = 0.18; M = 0.06; U = 0.77). However, positive items showed a higher percentage of explained method variance than trait variance—i.e., the method effect was stronger than the trait effect—in Greece (T = 0.05; M = 0.45; U = 0.50), Chile (T = 0.18; M = 0.30; U = 0.53), Brazil (T = 0.07; M = 0.29; U = 0.64), United States (T = 0.17; M = 0.23; U = 0.60), and Iran (T = 0.15; M = 0.21; U = 0.64). Negative items seemed to be particularly affected by method effects, because they showed a higher percentage of explained method variance than trait variance—i.e., negative items showed a greater method effect than trait effect—in all the samples: Korea (T = 0.03; M = 0.51; U = 0.47), United Kingdom (T = 0.06; M = 0.47; U = 0.47), Brazil (T = 0.12; M = 0.36; U = 0.52), Chile (T = 0.08; M = 0.33; U = 0.59), Greece (T = 0.21; M = 0.33; U = 0.45), United States (T = 0.14; M = 0.32; U = 0.54), Spain (T = 0.12; M = 0.26; U = 0.61), Japan (T = 0.10; M = 0.24; U = 0.65), Iran (T = 0.05; M = 0.23; U = 0.72), Saudi Arabia (T = 0.09; M = 0.18; U = 0.74), and Egypt (T = 0.08; M = 0.16; U = 0.76). Finally, the uniqueness term (U)—i.e., the percentage of unexplained variance—from positive and negative items was similar across the samples.

**Table 4:** Averaged variance components for the SCS in the MTMM approach.

	Br			Ch			Gr			Sp			UK			US		
	T	M	U	T	M	U	T	M	U	T	M	U	T	M	U	T	M	U
SK+	0.06	0.33	0.61	0.13	0.42	0.45	0.10	0.44	0.46	0.48	0.02	0.50	0.31	0.38	0.31	0.22	0.21	0.57
SK-	0.29	0.23	0.48	0.13	0.25	0.62	0.24	0.33	0.43	0.12	0.26	0.62	0.08	0.48	0.44	0.17	0.27	0.56
CH+	0.11	0.21	0.68	0.26	0.18	0.56	0.03	0.45	0.52	0.15	0.28	0.57	0.45	0.17	0.38	0.27	0.16	0.57
CH-	0.03	0.46	0.51	0.01	0.39	0.60	0.24	0.31	0.45	0.12	0.29	0.59	0.06	0.44	0.50	0.01	0.40	0.59
MI+	0.03	0.33	0.64	0.14	0.29	0.57	0.02	0.47	0.51	0.41	0.01	0.58	0.28	0.23	0.50	0.01	0.33	0.66
MI-	0.03	0.39	0.58	0.11	0.34	0.55	0.16	0.36	0.48	0.13	0.24	0.63	0.05	0.48	0.48	0.24	0.29	0.47

	**Ir**			**SA**			**Eg**			**Ko**			**Ja**		
	T	M	U	T	M	U	T	M	U	T	M	U	T	M	U

SK+	0.03	0.49	0.48	0.18	0.05	0.77	0.20	0.06	0.74	0.43	0.12	0.45	0.23	0.10	0.67
SK-	0.06	0.19	0.75	0.07	0.18	0.76	0.04	0.12	0.84	0.01	0.49	0.50	0.09	0.17	0.74
CH+	0.15	0.07	0.78	0.19	0.04	0.77	0.10	0.09	0.81	0.35	0.19	0.46	0.30	0.06	0.64
CH-	0.04	0.27	0.69	0.13	0.15	0.72	0.13	0.16	0.71	0.04	0.54	0.42	0.11	0.26	0.63
MI+	0.26	0.08	0.66	0.26	0.04	0.70	0.23	0.02	0.75	0.52	0.08	0.40	0.24	0.17	0.59
MI-	0.05	0.24	0.71	0.07	0.20	0.74	0.07	0.19	0.74	0.03	0.49	0.48	0.11	0.30	0.59

*Values are averaged components of variance and can be interpreted as percentages of explained variance. T, trait variance; M, method variance; U, uniqueness term. SK+, self-kindness positive items; SK-, self-kindness negative items; CH+, common humanity positive items; CH-, common humanity negative items; MI+, mindfulness positive items; MI-, mindfulness negative items; Br, Brazil; Ch, Chile; Gr, Greece; Sp, Spain; United Kingdom, United Kingdom; US, United States; Ir, Iran; SA, Saudi Arabia; Eg, Egypt; Ko, Korea; Ja, Japan.*

### Common Variance Among Traits and Between Methods

We found a great variety of common variance values among trait factors and between method components in the study samples (Table 5). The averaged percentage of common variance among the trait factors of self-kindness, common humanity and mindfulness was very diverse, pointing to differences in terms of their divergence/convergence as a multiple/unitary construct of self-compassion: Chile (*R*^2^ = 0.01), United States (*R*^2^ = 0.02), Brazil (*R*^2^ = 0.27), United Kingdom (*R*^2^ = 0.39), Egypt (*R*^2^ = 0.48), Saudi Arabia (*R*^2^ = 0.61), Iran (*R*^2^ = 0.63), Greece (*R*^2^ = 0.65), Korea (*R*^2^ = 0.69), Spain (*R*^2^ = 0.72), and Japan (*R*^2^ = 0.82). On the other hand, the percentage of common variance between the positive and the negative method factors also differed—ranging from *R*^2^ = 0.09 (Iran) to *R*^2^ = 0.90 (Saudi Arabia)—with consequences for the feasibility of separating/merging the positive and negative counterparts of the SCS questionnaire.

**Table 5:** Common variance among trait and between method SCS components.

	Br	Ch	Gr	Sp	UK	US	Ir	SA	Eg	Ko	Ja
**Trait components**					
SK ↔ CH	0.04	0.01	0.55	0.74	0.30	0.02	0.49	0.44	0.62	0.56	0.88
SK ↔ MI	0.70	0.01	0.54	0.67	0.22	0.01	0.61	0.46	0.12	0.83	0.88
MI ↔ CH	0.07	0.01	0.85	0.74	0.64	0.03	0.79	0.94	0.71	0.67	0.69
**Method components**					
Positive ↔ Negative	0.29	0.44	0.35	0.24	0.62	0.56	0.09	0.90	0.85	0.28	0.26

*Values are determination coefficients (*R*^2^) which can be interpreted as the percentage of common variance; SK, CH, and MI are self-kindness, common humanity and mindfulness, integrating the positive and negative items after removing method effects. Positive and Negative are positive and negative method components; Br, Brazil; Ch, Chile; Gr, Greece; Sp, Spain; United Kingdom, United Kingdom; US, United States; Ir, Iran; SA, Saudi Arabia; Eg, Egypt; Ko, Korea; Ja, Japan.*

### Relationships Between the MTMM Trait Parameters and Cultural Values

Data of the analyses developed at the country level (*n* = 11) on cultural values are shown in Table 6. As can be seen, we observed a large range of VSM scores among countries in all the cultural dimensions. When analyzing the relationships between the SCS trait parameters obtained by means of the MTMM approach—as referred to in the preceding sections—and the normative scores of the VSM cultural values, we observed some salient relationships for each MTMM parameter (Table 6). For instance, the percentage of explained variance in the positive items from the self-compassion trait factors was significantly related to the long-term orientation cultural value, with strong effects (*r*_s_ = 0.62; *p* = 0.042). The percentage of explained variance in the negative items from the self-compassion trait factors, although not statistically significantly related to individualism, nevertheless showed intermediate effects (*r*_s_ = 0.40; *p* = 0.228). Finally, the degree of convergence among the self-compassion trait factors—in terms of the percentage of common variance among them—was significantly and negatively related to indulgence, with strong effects (*r*_s_ = -0.65; *p* = 0.030). These were the most salient relationships between each SCS trait parameter and the VSM cultural values, although other non-significant but moderate relationships were also found (Table 6).

**Table 6:** Hofstede’s cultural values and relationships with the SCS trait parameters^†^.

	Individualism	Masculinity	Power distance	Long-term	Uncertainty	Indulgence
**Country values**					
Brazil	38	49	69	44	76	59
Chile	23	28	63	31	86	68
Greece	35	57	60	45	100	50
Spain	51	42	57	48	86	44
United Kingdom	89	66	35	51	35	69
USA	91	62	40	26	46	68
Iran	41	43	58	14	59	40
Saudi Arabia	25	60	95	36	80	52
Egypt	25	45	70	7	80	4
Korea	18	39	60	100	85	29
Japan	46	95	54	88	92	42
**Descriptive data**					
Mn	43.82	53.27	60.09	44.55	75.00	47.73
SD	25.00	17.89	15.78	28.21	20.01	19.48
Range	18–91	28–95	35–95	7–100	35–100	4–69
**Parameter correlations**					
Positive	-0.03	-0.06	-0.27	0.62^∗^	0.03	-0.13
Negative	0.40	0.38	-0.06	-0.05	0.29	0.30
Convergence	0.01	0.06	-0.17	0.56	0.55	-0.65^∗^

*^†^Analyses developed at the country level (*n* = 11), using Hofstede’s norms as input data of cultural values (Hofstede et al., 2010). Mn, Mean; SD, standard deviation; Range, lowest–highest values. Positive, percentage of SCS trait variance explained by positive items. Negative, percentage of SCS trait variance explained by negative items. Convergence, percentage of common variance among the SCS trait factors. Correlation values are Spearman’s coefficients. ^∗^*p* < 0.05.*

## Discussion

This study contributes to our understanding of how the self-compassion construct manifests itself in different cultures, and how it is related to different cultural values. Previous SCS validation studies have taken a range of approaches and found: six-correlated factors (Neff et al., 2008; Lee and Lee, 2010; Hupfeld and Ruffieux, 2011; Azizi et al., 2013; Arimitsu, 2014; Garcia-Campayo et al., 2014; Petrocchi et al., 2014; Williams et al., 2014; Mantzios et al., 2015; Bento et al., 2016; de Souza and Hutz, 2016); a second-order factor (Neff, 2003a; Chen et al., 2011; Castilho et al., 2015; Benda and Reichová, 2016; Dundas et al., 2016); a single bifactor (Kotsou and Leys, 2016; Tóth-Király et al., 2017; Veneziani et al., 2017; Cleare et al., 2018; Neff et al., 2018); two first-order factors (López et al., 2015; Costa et al., 2016); two second-order factors (Pfattheicher et al., 2017); two independent bifactors (Brenner et al., 2017; Coroiu et al., 2018); two correlated bifactors (Halamová et al., 2018), three positive factors alongside a general negative factor (Zeng et al., 2016), and a split of the positive and negative halves (Montero-Marin et al., 2016a; Muris and Petrocchi, 2017; Muris et al., 2018). We took a new perspective, asking “How does SCS’ mixture of positively- and negative-keyed items contributes to the self-compassion construct in different cultures?” and “How does the convergence of the dimensions of self-compassion relate to cultural values?”

A confirmatory MTMM approach was taken to decompose the overall SCS variance into trait, method and uniqueness, conceiving the method factor as the positive/negative valence of the items. This enables us to establish if the valence of the items helps us to better understand how the self-compassion construct is built by the SCS, and whether this varies by culture. To date researchers often have found six-factors composed of three positive and negative factors (Neff et al., 2008; Lee and Lee, 2010; Hupfeld and Ruffieux, 2011; Azizi et al., 2013; Arimitsu, 2014; Garcia-Campayo et al., 2014; Petrocchi et al., 2014; Williams et al., 2014; Bento et al., 2016; de Souza and Hutz, 2016; Mantzios et al., 2015), which sometimes have been subsumed into higher order structures (Neff, 2003a; Chen et al., 2011; Castilho et al., 2015; Benda and Reichová, 2016; Dundas et al., 2016; Kotsou and Leys, 2016; Tóth-Király et al., 2017; Veneziani et al., 2017; Cleare et al., 2018; Neff et al., 2018). This pattern of positive/negative factors is not unique to the SCS but has also been found in other psychological measures such as the Positive and Negative Affect Schedule (PANAS), which also presents structural ambiguities in its functioning (Merz et al., 2013) that could probably be determined by some contextual aspects—e.g., the presentation form used (Seib-Pfeifer et al., 2017). There is enough evidence in the psychometric literature that the use of positive and negative items increases method variance, which makes method factors appear that are not really associated with the traits of interest (Williams et al., 2002).

After discarding possible method effects by using the MTMM model, in the majority of cases (although not in all of them), we observed that the positively valenced items, compared with the negative ones (which suffered more from method effects), were better explained by the corresponding trait factors of self-compassion. Therefore, it might be proposed that measurement of self-compassion only through positive items, would mitigate unwanted effects resulting from emerging artifact components (Spector et al., 1997; van Sonderen et al., 2013). This idea is consistent with the findings of Swain et al. (2008), who reported that respondents tend to answer more accurately with items that reflect their experience than with items that describe the opposite of their experience, as captured in reversed items. A consequence of the use of reversed items can be the identification of two unipolar concepts where there is only one, committing the methodological error of reification, as described with other health-related questionnaires (Hankins, 2008). However, we observed the percentage of trait explained variance in the positive/negative SCS items was distributed with relative variability among the different study samples, something that according to our exploratory hypotheses, might be explained by the influence of respondents’ cultural background and values.

The study findings suggest that the long-term orientation cultural value was strongly and directly related to the percentage of explained variance in the positive items, once released of method effects. In general, the long-term orientation cultural value stands for the fostering of virtues oriented toward future rewards, such as financial prudence and effort in the pursuit of education and goals, with humility (Hofstede et al., 2010). It has been previously found that the levels of the positive, but not the negative, SCS facets are directly correlated with the levels of positive attitudes toward challenges during learning processes through “desirable difficulties” learning strategies, maybe due to less fear of failure, higher control beliefs, and mastery goals (Wagner et al., 2017). Thus, in the context of our study, we could venture to suppose that the long-term orientation cultural value might motivate positive actions to support the growth and flourishing of self-competence, helping people to cope with situations that threaten their adequacy through warmth and interconnectedness (Neff, 2003a,b; Neff et al., 2005; Cozolino, 2007; Gilbert, 2010, 2014). However, we must not lose sight of the fact that the level of certain characteristic is not the same than the importance of that characteristic in explaining a broader construct, as it has been referred above.

Long-term orientation has been considered essential in the civilization process and has appeared in previous research as a social value associated with deferment of gratification and shame (Hofstede et al., 2010). This is easy to understand if we consider that children have to learn a considerable amount of self-control in order to be accepted as civilized persons in the different human societies. Interestingly, we also found that long-term orientation, uncertainty avoidance, and indulgence were associated with the degree of convergence between the general self-compassion traits. Indulgence—as the opposite pole of restraint—evidenced strong inverse correlations with the degree of convergence of the self-compassion traits. Thus, our results suggest that self-compassion traits might be close to the point of being essentially the same construct, in those societies that suppresses immediate gratification of desires and regulates them by means of social norms, in contexts that promote self-competence and avoiding uncertainty through a long-term orientation (Hofstede et al., 2010). This pattern of convergent self-compassion traits mainly built by the contribution of the positive SCS items, in cultural contexts characterized by high long-term orientation, uncertainty avoidance, and restraint, was observed in the Korean and Japanese samples. The possible influence of Eastern philosophy that emphasizes compassion practices (Strauss et al., 2016), would be worthy of investigation in future research. Within these practices, compassion is seen as a result of wisdom, which is embedded in an ethical background mediated by the selfless intention of freeing from suffering, and in which the duality of self-others is relativized (Strauss et al., 2016; Gu et al., 2017), something that might result in a more integrated self-compassion construct.

On the other hand, the percentage of explained trait variance in the negative items once freed of method effects was not significantly related to cultural values, although we found moderate effects with individualism and masculinity. Individualism characterizes social contexts in which subjects are expected to be independent and take care primarily of only themselves, with no dependence or loyalty to groups or family members, and thus the ties between individuals are loose (Hofstede, 2001; Hofstede et al., 2010). Meanwhile, masculinity represents a contextual preference for assertiveness, personal initiative, competition, and domination in social life based on achievements and material success (Hofstede, 2001; Hofstede et al., 2010). This combination of the cultural values of individualism and masculinity, might create a framework characterized by a competitive system of motivational and interpersonal relating, with prominent social ranking (Gilbert, 2014). Within this system, social comparison exerts a determining role, activating self-referent cognitive processing, and in case of performance difficulties or failures, resulting in fear of defeat and isolation. These concerns could foreground self-critical attitudes, as they are described in the SCS negative items, when configuring the self-compassion construct (Neff et al., 2005; Gilbert, 2009; Montero-Marin et al., 2016a). The case of USA was an example of this pattern in which there were high levels of individualism and masculinity, the negative items more prominently explained the self-compassion traits, and in which there was a disaggregated self-compassion construct. It has been suggested that the way in which individualism and masculinity place the person in relation to the group, and the consequent difficulties in cooperation and altruism that might emerge (Henrich et al., 2005), could shape differences in the construct of compassion (Goetz et al., 2010). We think it might specifically contribute to a greater relative contribution of the negative SCS counterpart when shaping the construct, and also a disaggregation among the components of self-compassion, as we have found in the USA example. It would be interesting to investigate whether/to what extent the USA values of self-determination effort and self-examination underlie and feed these tendencies (Ehrenreich, 2009).

One other cultural dimension that might shed light onto the cross-cultural issue of the self-compassion construct structure is “dialecticism” (Peng and Nisbett, 1999). Dialecticism refers to the way in which individuals perceive conflicting concepts and develop a complete point of view from them. For example, Eastern cultures seem to retain basic elements of opposing perspectives by seeking something like a “middle way,” and Western cultures differentiate the polarized contradictory perspectives in an effort to be more positioned at one of the extremes (Peng and Nisbett, 1999). This dialecticism is also observed at an individual, emotional level, with people belonging to Eastern cultures more likely to experience a balance of positive and negative emotions, while people belonging to Western cultures being more prone to experience predominantly positive or predominantly negative emotions. Interestingly, we have observed that countries that showed higher self-compassion trait convergence coincide with countries that generally tend to avoid extremes. However, whether/to what exent dialecticism as a cultural value plays a significant role in the structure of self-compassion—in terms of the relative contribution of positively and negatively worded statements, and the convergence of their different components—is unknown, and a question that should be clarified through future research. Curiously, the idiosincasy of the SCS questionnaire, being built as it is through items tapping opposite poles, offers a good opportunity to investigate the possible effect of dialecticism on the self-compassion construct itself.

In general, and similarly across samples, method factors had a greater prominence in the negatively valenced items. It has been suggested the personality traits of neuroticism and self-criticism underlie to a large degree endorsement of the negative SCS items (Mills et al., 2007; Pfattheicher et al., 2017). Something similar might be occurring with the positive SCS items, in terms of positive affect (Phillips and Ferguson, 2013). It has also been suggested that SCS items, especially those related to self-kindness, are open to interpretation, and do not tell us about how self-compassion is enacted in everyday life (Mantzios and Egan, 2017). There is the element of self-care that needs to be aligned to self-kindness, and if this is not the case, self-kindness might become self-indulgence. In the present study, and due to the nature of the generated matrices by the complete MTMM model, we were not able to analyze the possible multidimensionality of method effects (Marsh and Grayson, 1995), in which neuroticism, self-criticism, affectivity and self-indulgence might be included (it is necessary to highlight that we studied indulgence as a contextual value of influence, but we did not use self-indulgence at an individual level, as an enacted behavior). This brings a possible confusion in considering as method variance some amount of variance that in fact could be coming from other individual psychological traits—although our design protects us to a certain extent from this problem because traits and method factors were not correlated (Bagozzi, 1993; Byrne and Goffin, 1993). We have also seen that the percentage of common variance shared between the positive and negative method factors of the SCS largely differed across samples. This difference may be one of the reasons for inconsistency in the SCS factorial structure across studies, obtaining a total score for self-compassion emerging from the opposite counterparts (Neff, 2003a), but also the deconstruction of the questionnaire into two halves (MacBeth and Gumley, 2012). Thus, further research with the method part of the MTMM more defined is required to understand the origins of these differences and whether affects, self-indulgence, neuroticism and self-criticism might be differentially materialized across cultures in SCS method factors.

The main strength of this study was the comprehensiveness of the cross-cultural design which allowed us to compare the sources of variability of the evaluated construct through samples from 11 different countries and 5 geographical regions of influence, with 8 distinct languages, cultural backgrounds, and a large sample size. The homogeneity of the samples used, being composed in each case of undergraduate university students, on the one hand, facilitated the comparability among them as it is recommended when comparing cultural values (Hofstede, 2001; Hofstede et al., 2010), but on the other hand, results in samples representing participants at a particular cultural level, which is also an important limitation. Likewise, the flexibility of the ULS data analysis method used allowed us to reach accurate and robust parameter estimates with no restrictive assumptions regarding the distribution of data, but at the cost of not being able to contrast possible differences in the parametres among samples by using *p*-values, invariance analyses, and other fit-indices. Moreover, in some cases small and similar factor loadings were found, perhaps as a consequence of having forced the factorial weights to be different during the iterative process of parameter estimation, so more research is needed to ensure the stability of results (Kenny and Kashy, 1992).

In addition, because the structure of the SCS and the definition of compassion/self-compassion is still being debated, we did not use additional measures to evaluate convergent and discriminant validity. As a result, we interpreted the higher-order positive and negative method factors as representing method effects, but this should be contrasted with other measures to determine, e.g., whether underlying the negatively worded items there could be a method effect or lack of self-compassion. Testing of convergent and discriminant validity would also be needed to ensure that labeling the factors in similar ways, across cultures, is valid and would help with interpreting the differences in trait and method variance noted in the present study. The novelty of the MTMM procedure application, which makes possible studying both method and trait variance at the same time, offers a new approach to studying the SCS structure. However, this is at the cost of making it difficult to directly compare results obtained in previous research. More studies are needed that use samples drawn from more diverse populations and also other MTMM model specifications—e.g., introducing covariances among trait and method factors to know the degree of dependence between them. Finally, we found moderate relationships between the MTMM parameters and the cultural values that resulted in non-significant results, which may be due to there being only 11 different samples at the country level, and therefore, the statistical capability to test the corresponding relationship may not have been powered to obtain significant results with mild effects. Nevertheless, the effort needed to obtain a greater number of samples at the country-level is enormous, and we should consider the present work as exploratory and hypothesis generating in a fascinating area not free of controversy and still in development (Strauss et al., 2016).

## Conclusion

Our research suggest distinct conceptualizations of the self-compassion construct depending on the contextual values in which subjects are immersed, which is consistent with the idea that the way people think, feel and behave tends to be congruent with the values shared by members of their own community, and that might be established by means of a collective mental programming through socialization processes (Hofstede, 2001; Hofstede et al., 2010). We have observed that the positive items of the SCS are more important than the negative items in operationalizing the self-compassion construct, and thus measuring self-compassion-relevant attitudes and behaviors in positive terms could make a greater contribution to capturing the components comprising the construct. However, our study suggests that the degree to which this is so might depend on how dominant values embedded in the cultural background shape the way self-compassion is manifest. For example, long-term orientation and individualism might be influencing the salience of the positive and negative items, respectively. In addition, the convergence between SCS traits, and hence the extent to which they might be regarded as reflecting aspects of a broader self-compassion construct, seems to differ according to the indulgence-restraint cultural dimension. We consider this work on the relationships between the self-compassion construct and predominant cultural values to be exploratory and hypothesis-generating. Nonetheless, it offers numerous avenues to clarify, extend and refine our understanding of self-compassion and its role in alleviating suffering around the world.

## Ethics Statement

The study protocol was approved by the ethical review board of the regional health authority of Aragon (CEICA), Spain (PI17/099). Data from all samples were originally obtained with the written informed consent of the participants, and the specific ethical requirements of each place of origin were considered. The data files analyzed in the present study will be available upon request to be used for replication, and authors will send the referred materials under reasonable conditions. All procedures contributing to the present study were performed in accordance with the ethical standards as prescribed in the Declaration of Helsinki and its later amendments, the Declaration of Madrid of the World Psychiatric Association, and the established requirements for manuscripts submitted to Bio-medical journals.

## Author Contributions

JM-M, WK, CC, and JG-C designed the study. JM-M executed the study and developed the data analyses. JM-M and CC were in charge of the first writing of the manuscript. WK, JG, RB, and JG-C collaborated in the writing of the manuscript. JM-M, AA-A, SA, CA-V, NG, ZC, M-SK, MM, DRdS, LSL, AT, PW, AY, EY, and JG-C supplied the different study samples. All the authors revised critically and approved the final version of the manuscript. The place of the authors whose main contribution were samples of participants has been entered in alphabetical order. All the authors collaborated in the editing of the final manuscript version.

## Conflict of Interest Statement

The authors declare that the research was conducted in the absence of any commercial or financial relationships that could be construed as a potential conflict of interest.
