# Supplementary material for: Self-Compassion and Cultural Values: A Cross-Cultural Study of Self-Compassion Using a Multitrait-Multimethod (MTMM) Analytical Procedure
Source: Front Psychol. 2018 Dec 21;9:2638. doi: 10.3389/fpsyg.2018.02638 (PMC6308155; doi:10.3389/fpsyg.2018.02638)
Supplement: Supplementary file 1 [file Table_1.DOCX]

**Additional File 1: Trait and method variance components for the SCS items in the MTMM approach**

|  |  |  |  | **Br** |  |  |  | **Ch** |  |  |  | **Gr** |  |  |  | **Sp** |  |  |  | **UK** |  |  |  | **US** |  |  |  | **Ir** |  |  |  | **SA** |  |  |  | **Eg** |  |  |  | **Ko** |  |  |  | **Ja** |  |  |
| --- | --- | --- | --- | --- | --- | --- | --- | --- | --- | --- | --- | --- | --- | --- | --- | --- | --- | --- | --- | --- | --- | --- | --- | --- | --- | --- | --- | --- | --- | --- | --- | --- | --- | --- | --- | --- | --- | --- | --- | --- | --- | --- | --- | --- | --- | --- |
|  | **Items** |  | **T** | **M** | **U** |  | **T** | **M** | **U** |  | **T** | **M** | **U** |  | **T** | **M** | **U** |  | **T** | **M** | **U** |  | **T** | **M** | **U** |  | **T** | **M** | **U** |  | **T** | **M** | **U** |  | **T** | **M** | **U** |  | **T** | **M** | **U** |  | **T** | **M** | **U** |  |
| SK+ | SCS5 |  | 0.01 | 0.36 | 0.63 |  | 0.14 | 0.35 | 0.51 |  | 0.01 | 0.41 | 0.58 |  | 0.29 | 0.02 | 0.69 |  | 0.55 | 0.14 | 0.31 |  | 0.06 | 0.09 | 0.85 |  | 0.12 | 0.15 | 0.73 |  | 0.19 | 0.11 | 0.70 |  | 0.33 | 0.04 | 0.63 |  | 0.49 | 0.12 | 0.39 |  | 0.25 | 0.06 | 0.69 |  |
|  | SCS12 |  | 0.01 | 0.55 | 0.44 |  | 0.19 | 0.33 | 0.48 |  | 0.07 | 0.42 | 0.51 |  | 0.53 | 0.01 | 0.46 |  | 0.46 | 0.26 | 0.28 |  | 0.29 | 0.18 | 0.53 |  | 0.01 | 0.35 | 0.64 |  | 0.25 | 0.04 | 0.71 |  | 0.14 | 0.03 | 0.83 |  | 0.55 | 0.13 | 0.32 |  | 0.34 | 0.01 | 0.65 |  |
|  | SCS19 |  | 0.02 | 0.26 | 0.72 |  | 0.19 | 0.38 | 0.43 |  | 0.08 | 0.45 | 0.47 |  | 0.48 | 0.01 | 0.51 |  | 0.37 | 0.31 | 0.32 |  | 0.37 | 0.22 | 0.41 |  | 0.02 | 0.36 | 0.62 |  | 0.27 | 0.02 | 0.71 |  | 0.49 | 0.04 | 0.47 |  | 0.50 | 0.09 | 0.41 |  | 0.28 | 0.03 | 0.69 |  |
|  | SCS23 |  | 0.19 | 0.20 | 0.61 |  | 0.07 | 0.52 | 0.41 |  | 0.20 | 0.45 | 0.35 |  | 0.56 | 0.04 | 0.40 |  | 0.02 | 0.71 | 0.27 |  | 0.11 | 0.30 | 0.59 |  | 0.13 | 0.22 | 0.65 |  | 0.08 | 0.06 | 0.86 |  | 0.02 | 0.18 | 0.80 |  | 0.21 | 0.05 | 0.74 |  | 0.04 | 0.23 | 0.73 |  |
|  | SCS26 |  | 0.08 | 0.28 | 0.64 |  | 0.05 | 0.52 | 0.43 |  | 0.12 | 0.46 | 0.42 |  | 0.55 | 0.01 | 0.44 |  | 0.15 | 0.49 | 0.36 |  | 0.28 | 0.25 | 0.47 |  | 0.12 | 0.27 | 0.61 |  | 0.10 | 0.02 | 0.88 |  | 0.03 | 0.01 | 0.96 |  | 0.38 | 0.22 | 0.40 |  | 0.23 | 0.19 | 0.58 |  |
| SK- | SCS1 |  | 0.27 | 0.01 | 0.72 |  | 0.09 | 0.15 | 0.76 |  | 0.24 | 0.37 | 0.39 |  | 0.05 | 0.21 | 0.74 |  | 0.11 | 0.52 | 0.37 |  | 0.10 | 0.28 | 0.62 |  | 0.02 | 0.19 | 0.79 |  | 0.01 | 0.08 | 0.91 |  | 0.01 | 0.10 | 0.89 |  | 0.03 | 0.49 | 0.48 |  | 0.06 | 0.16 | 0.78 |  |
|  | SCS8 |  | 0.35 | 0.26 | 0.39 |  | 0.19 | 0.27 | 0.54 |  | 0.42 | 0.21 | 0.37 |  | 0.06 | 0.28 | 0.66 |  | 0.08 | 0.52 | 0.40 |  | 0.20 | 0.20 | 0.60 |  | 0.01 | 0.22 | 0.77 |  | 0.03 | 0.15 | 0.82 |  | 0.04 | 0.17 | 0.79 |  | 0.01 | 0.42 | 0.57 |  | 0.21 | 0.02 | 0.77 |  |
|  | SCS11 |  | 0.28 | 0.28 | 0.44 |  | 0.05 | 0.29 | 0.66 |  | 0.10 | 0.35 | 0.55 |  | 0.17 | 0.28 | 0.55 |  | 0.05 | 0.40 | 0.55 |  | 0.26 | 0.31 | 0.43 |  | 0.12 | 0.11 | 0.77 |  | 0.20 | 0.16 | 0.64 |  | 0.03 | 0.20 | 0.77 |  | 0.01 | 0.55 | 0.44 |  | 0.05 | 0.17 | 0.78 |  |
|  | SCS16 |  | 0.42 | 0.34 | 0.24 |  | 0.20 | 0.21 | 0.59 |  | 0.17 | 0.46 | 0.37 |  | 0.18 | 0.38 | 0.44 |  | 0.03 | 0.62 | 0.35 |  | 0.17 | 0.27 | 0.56 |  | 0.04 | 0.29 | 0.67 |  | 0.05 | 0.31 | 0.64 |  | 0.03 | 0.10 | 0.87 |  | 0.01 | 0.52 | 0.47 |  | 0.08 | 0.36 | 0.56 |  |
|  | SCS21 |  | 0.13 | 0.28 | 0.59 |  | 0.11 | 0.35 | 0.54 |  | 0.26 | 0.28 | 0.46 |  | 0.12 | 0.15 | 0.73 |  | 0.14 | 0.36 | 0.50 |  | 0.11 | 0.31 | 0.58 |  | 0.12 | 0.11 | 0.77 |  | 0.05 | 0.18 | 0.77 |  | 0.10 | 0.01 | 0.89 |  | 0.01 | 0.46 | 0.53 |  | 0.07 | 0.12 | 0.81 |  |
| CH+ | SCS3 |  | 0.05 | 0.33 | 0.62 |  | 0.07 | 0.24 | 0.69 |  | 0.04 | 0.40 | 0.56 |  | 0.04 | 0.21 | 0.75 |  | 0.35 | 0.17 | 0.48 |  | 0.29 | 0.13 | 0.58 |  | 0.20 | 0.04 | 0.76 |  | 0.07 | 0.01 | 0.92 |  | 0.14 | 0.09 | 0.77 |  | 0.30 | 0.08 | 0.62 |  | 0.31 | 0.03 | 0.66 |  |
|  | SCS7 |  | 0.16 | 0.14 | 0.70 |  | 0.53 | 0.01 | 0.46 |  | 0.01 | 0.48 | 0.51 |  | 0.08 | 0.44 | 0.48 |  | 0.53 | 0.14 | 0.33 |  | 0.20 | 0.08 | 0.72 |  | 0.02 | 0.04 | 0.94 |  | 0.26 | 0.03 | 0.71 |  | 0.06 | 0.05 | 0.89 |  | 0.19 | 0.27 | 0.54 |  | 0.26 | 0.01 | 0.73 |  |
|  | SCS10 |  | 0.19 | 0.07 | 0.74 |  | 0.40 | 0.06 | 0.54 |  | 0.01 | 0.49 | 0.50 |  | 0.07 | 0.44 | 0.49 |  | 0.52 | 0.14 | 0.34 |  | 0.33 | 0.13 | 0.54 |  | 0.04 | 0.14 | 0.82 |  | 0.29 | 0.01 | 0.70 |  | 0.03 | 0.14 | 0.83 |  | 0.23 | 0.36 | 0.41 |  | 0.34 | 0.06 | 0.60 |  |
|  | SCS15 |  | 0.02 | 0.28 | 0.70 |  | 0.04 | 0.42 | 0.54 |  | 0.05 | 0.44 | 0.51 |  | 0.42 | 0.01 | 0.57 |  | 0.41 | 0.21 | 0.38 |  | 0.24 | 0.30 | 0.46 |  | 0.34 | 0.06 | 0.60 |  | 0.13 | 0.10 | 0.77 |  | 0.16 | 0.06 | 0.78 |  | 0.66 | 0.06 | 0.28 |  | 0.27 | 0.14 | 0.59 |  |
| CH- | SCS4 |  | 0.01 | 0.48 | 0.51 |  | 0.01 | 0.33 | 0.66 |  | 0.41 | 0.19 | 0.40 |  | 0.12 | 0.35 | 0.53 |  | 0.10 | 0.50 | 0.40 |  | 0.02 | 0.33 | 0.65 |  | 0.10 | 0.19 | 0.71 |  | 0.02 | 0.18 | 0.80 |  | 0.01 | 0.21 | 0.78 |  | 0.05 | 0.55 | 0.40 |  | 0.14 | 0.22 | 0.64 |  |
|  | SCS13 |  | 0.01 | 0.52 | 0.47 |  | 0.02 | 0.29 | 0.69 |  | 0.09 | 0.46 | 0.45 |  | 0.15 | 0.33 | 0.52 |  | 0.06 | 0.36 | 0.58 |  | 0.01 | 0.44 | 0.55 |  | 0.01 | 0.21 | 0.78 |  | 0.05 | 0.29 | 0.66 |  | 0.11 | 0.22 | 0.67 |  | 0.02 | 0.50 | 0.48 |  | 0.07 | 0.30 | 0.63 |  |
|  | SCS18 |  | 0.01 | 0.34 | 0.65 |  | 0.01 | 0.42 | 0.57 |  | 0.23 | 0.29 | 0.48 |  | 0.06 | 0.22 | 0.72 |  | 0.04 | 0.45 | 0.51 |  | 0.01 | 0.55 | 0.44 |  | 0.01 | 0.31 | 0.68 |  | 0.34 | 0.01 | 0.65 |  | 0.36 | 0.01 | 0.63 |  | 0.01 | 0.55 | 0.44 |  | 0.10 | 0.10 | 0.80 |  |
|  | SCS25 |  | 0.08 | 0.49 | 0.43 |  | 0.01 | 0.53 | 0.46 |  | 0.23 | 0.28 | 0.49 |  | 0.16 | 0.24 | 0.60 |  | 0.05 | 0.44 | 0.51 |  | 0.01 | 0.29 | 0.70 |  | 0.03 | 0.38 | 0.59 |  | 0.07 | 0.17 | 0.76 |  | 0.05 | 0.19 | 0.76 |  | 0.08 | 0.56 | 0.36 |  | 0.14 | 0.41 | 0.45 |  |
| MI+ | SCS9 |  | 0.04 | 0.37 | 0.59 |  | 0.34 | 0.15 | 0.51 |  | 0.02 | 0.35 | 0.63 |  | 0.35 | 0.01 | 0.64 |  | 0.21 | 0.15 | 0.64 |  | 0.02 | 0.16 | 0.82 |  | 0.25 | 0.01 | 0.74 |  | 0.31 | 0.03 | 0.66 |  | 0.33 | 0.01 | 0.66 |  | 0.41 | 0.03 | 0.56 |  | 0.34 | 0.01 | 0.65 |  |
|  | SCS14 |  | 0.05 | 0.44 | 0.51 |  | 0.17 | 0.33 | 0.50 |  | 0.01 | 0.55 | 0.44 |  | 0.49 | 0.01 | 0.50 |  | 0.36 | 0.25 | 0.41 |  | 0.01 | 0.38 | 0.61 |  | 0.58 | 0.02 | 0.40 |  | 0.16 | 0.02 | 0.82 |  | 0.11 | 0.01 | 0.88 |  | 0.62 | 0.04 | 0.34 |  | 0.24 | 0.08 | 0.68 |  |
|  | SCS17 |  | 0.01 | 0.23 | 0.76 |  | 0.02 | 0.40 | 0.58 |  | 0.01 | 0.58 | 0.41 |  | 0.45 | 0.01 | 0.54 |  | 0.29 | 0.30 | 0.41 |  | 0.01 | 0.35 | 0.64 |  | 0.12 | 0.19 | 0.69 |  | 0.25 | 0.01 | 0.74 |  | 0.30 | 0.01 | 0.69 |  | 0.64 | 0.04 | 0.32 |  | 0.18 | 0.38 | 0.44 |  |
|  | SCS22 |  | 0.01 | 0.26 | 0.72 |  | 0.01 | 0.27 | 0.72 |  | 0.02 | 0.38 | 0.60 |  | 0.34 | 0.02 | 0.64 |  | 0.24 | 0.21 | 0.55 |  | 0.01 | 0.44 | 0.55 |  | 0.10 | 0.08 | 0.82 |  | 0.30 | 0.09 | 0.61 |  | 0.19 | 0.04 | 0.77 |  | 0.41 | 0.23 | 0.36 |  | 0.21 | 0.19 | 0.60 |  |
| MI- | SCS2 |  | 0.02 | 0.41 | 0.57 |  | 0.03 | 0.44 | 0.53 |  | 0.15 | 0.46 | 0.39 |  | 0.22 | 0.35 | 0.43 |  | 0.03 | 0.74 | 0.23 |  | 0.34 | 0.20 | 0.46 |  | 0.01 | 0.21 | 0.78 |  | 0.05 | 0.26 | 0.69 |  | 0.22 | 0.31 | 0.47 |  | 0.05 | 0.52 | 0.43 |  | 0.05 | 0.45 | 0.50 |  |
|  | SCS6 |  | 0.06 | 0.37 | 0.57 |  | 0.01 | 0.40 | 0.59 |  | 0.23 | 0.29 | 0.48 |  | 0.11 | 0.28 | 0.61 |  | 0.05 | 0.46 | 0.49 |  | 0.17 | 0.31 | 0.52 |  | 0.05 | 0.27 | 0.68 |  | 0.04 | 0.29 | 0.67 |  | 0.03 | 0.29 | 0.68 |  | 0.03 | 0.49 | 0.48 |  | 0.14 | 0.26 | 0.60 |  |
|  | SCS20 |  | 0.01 | 0.45 | 0.54 |  | 0.28 | 0.15 | 0.57 |  | 0.18 | 0.31 | 0.51 |  | 0.03 | 0.10 | 0.87 |  | 0.06 | 0.37 | 0.57 |  | 0.42 | 0.18 | 0.40 |  | 0.12 | 0.15 | 0.73 |  | 0.01 | 0.17 | 0.82 |  | 0.03 | 0.12 | 0.85 |  | 0.01 | 0.40 | 0.59 |  | 0.18 | 0.13 | 0.69 |  |
|  | SCS24 |  | 0.01 | 0.33 | 0.66 |  | 0.11 | 0.38 | 0.51 |  | 0.06 | 0.37 | 0.57 |  | 0.14 | 0.21 | 0.65 |  | 0.05 | 0.33 | 0.62 |  | 0.03 | 0.48 | 0.49 |  | 0.02 | 0.33 | 0.65 |  | 0.18 | 0.06 | 0.76 |  | 0.01 | 0.03 | 0.96 |  | 0.02 | 0.55 | 0.43 |  | 0.06 | 0.35 | 0.59 |  |

Values are the components of variance of each item and can be interpreted as percentages of explained variance: T = Trait variance; M = Method variance; U = Uniqueness term.

SK+ (Self-Kindness, positive); SK- (Self-Kindness, negative); CH+ (Common Humanity, positive); CH- (Common Humanity, negative); MI+ (Mindfulness, positive); MI- (Mindfulness, negative).

Br = Brazil; Ch = Chile; Gr = Greece; Sp = Spain; UK = United Kindom; US = United States; Ir = Iran; SA = Saudi Arabia; Eg = Egypt; Ko = Korea; Ja = Japan.
